# COVID-19 Mass Vaccination Campaign: An International Comparison of Qatar With GCC Nations and Other Global Groups

**DOI:** 10.3389/ijph.2023.1605614

**Published:** 2023-04-04

**Authors:** Yasmin Ali Morad Abdullahi

**Affiliations:** Primary Healthcare Corporation (PHCC), Doha, Qatar

**Keywords:** public health, COVID-19, vaccination, leadership, mass campaign

## Abstract

**Objectives:** Mass vaccination has been a key component in the effort to control the COVID-19 pandemic. Different countries have formulated their mass vaccination campaigns in different ways and with different priorities, with varying results. This study focuses on the case of Qatar in comparison with regional neighbors in the Gulf Cooperation Community (GCC) and with global benchmarks (G7 and OECD nations) in terms of the deployment of its mass vaccination program.

**Methods:** Data on national vaccine administration and policy were obtained from Our World in Data and the Oxford COVID-19 Government Response Tracker for the period of 25 November 2020, when public vaccination first began to be implemented within the GCC, and June 2021, when Qatar’s mass vaccination campaign concluded. Factors compared cross-nationally included the total number of vaccine doses administered, doses administered per 100 population, the time taken to reach certain vaccination thresholds (5, 10, 25, 50, and 100 doses per 100 population), and policy regarding administration to specific priority groups. Cumulative vaccination rates were also compared graphically by date.

**Results:** A descriptive comparison of vaccination rates illustrated that there were similar aggregate patterns among the GCC, G7, and OECD groups of countries, and that there was a great deal of heterogeneity in the patterns of vaccination between countries within each of these groups. The mass vaccination program in Qatar outpaced the aggregate GCC, G7, and OECD groups.

**Conclusion:** There were large between-country differences in the speed of mass vaccination progress which did not appear to be directly explained by national wealth. It is suggested that administrative and program management factors could account for some of these differences.

## Introduction

The SARS-CoV-2 coronavirus was first described in December 2019 (1), and by 11 March 2020 the World Health Organization (WHO) had declared COVID-19 to be a global pandemic (2). As of May 2021, the pandemic had caused at least more than 150 million diagnosed illnesses and led to more than 3.2 million deaths (3). In addition to direct mortality, COVID-19 can cause other serious health effects, including neurological (4), cardiac (5), and respiratory symptoms (6) that may last for more than 6 months in a significant number of patients (7). When these impacts, as well as the indirect health effects brought about by social isolation and economic disruption, are taken into consideration, the full toll of the COVID-19 pandemic is likely to be many times higher than the aforementioned figure of 3.2 million. Although the development of therapies for treating the symptoms of the disease are of great importance, and advances in this area have undoubtedly played an important role (8), along with non-pharmaceutical interventions vaccination is considered to be the most important element in ending the pandemic by curtailing the spread of the virus (9). Since the first approval of vaccines for emergency use in Russia and China in August 2020, governments around the world have responded by organizing mass vaccination campaigns aimed at protecting their populations as quickly and efficiently as possible. Various governments have adopted different approaches to designing these programs, and have faced different challenges in implementing them, making cross-national comparison a useful tool for examining the potential efficacy of approaches that may be used in the future.

The purpose of this study was to examine the interim performance of Qatar’s COVID-19 mass vaccination program, in comparison with other programs in the GCC region and globally. The primary outcomes of interest examined were the rate of vaccine administration, the pace of expansion of vaccine availability to high-risk and general population groups, and the specific vaccines in use in each country.

### Barriers to Implementation

At the level of planning and implementation, there are several important barriers that vary between jurisdictions and that may have a substantial impact on the pace and success of a mass vaccination program. These logistical challenges include the need to obtain vaccine doses, the need to efficiently organize the personnel required to administer them, infrastructure requirements for proper vaccine storage and delivery, and the problem of vaccine hesitancy among the population. Major cross-national disparities in vaccine distribution are evident, particularly based on economic factors. Given high per dose costs for many vaccines, the ability to purchase a sufficient quantity of vaccine to cover an entire national population is beyond the budgetary means of many lower- and middle-income countries (10). Even among those with the means to pay, supply limitations and export controls in vaccine-producing countries may limit the ability to obtain sufficient doses (11). Infrastructure challenges include requirements for advanced refrigeration equipment for some vaccines, which are expensive and not readily available in all countries (12), as well as the number of trained medical personnel available to be deployed to administer vaccines (13). Skepticism among members of the population regarding the safety, efficacy, or importance of receiving the vaccine, a phenomenon known as vaccine hesitancy, is an additional challenge faced in the administration of mass vaccination programs and may act to slow the rate of vaccination even when sufficient resources and infrastructure for delivery exist (14).

Qatar is classified as a high-income developing economy by the United Nations (15) (in this context, “high income” is defined based solely on Gross National Income, whereas development level incorporates a broader context of historical and political factors, in addition to econometric ones). Its national healthcare infrastructure has developed extremely rapidly in recent decades (16). This may bring a mixture of advantages and disadvantages in the present situation; the health system has very modern technology capable of delivering vaccines to patients but may lack a reserve of trained healthcare personnel to meet the demand for administration.

### Vaccination Program Evaluation Metrics

Some of the key elements by which vaccination programs can be compared include the speed with which the overall population is vaccinated, the speed with which specific vulnerable populations are protected, and the types of vaccine administered. Some authors contend that a population may be considered to have attained herd immunity when approximately 70% have been vaccinated (17). According to a classical view of the dynamics of infection within a population, at this point, the rate of the spread of the virus is expected to be sufficiently attenuated that members of the population who are unable to be vaccinated (e.g., due to underlying immune conditions that may make vaccination dangerous or ineffective) are unlikely to be exposed in the community. Conversely, other researchers have made the case that herd immunity is not a realistic goal for COVID-19, because the specific properties of the virus and the mode by which it is impacted by vaccination (18). In spite of these reservations, achieving a 70% rate of vaccination has widely been considered one of the primary goals of any mass vaccination campaign.

Another important consideration is prioritization of specific groups that are especially vulnerable to COVID-19 infection, either due to high risk of exposure or to a high risk of developing serious symptoms once infected. For example, healthcare workers have experienced high rates of COVID-19 infection during the pandemic (19), so a focus on early vaccination within this group has the dual advantage of protecting individuals at high risk of becoming infected, as well as helping to minimize disruptions to the functioning of essential healthcare services (20). Similarly, older adults and people with chronic respiratory and cardiovascular comorbidities who have contracted COVID-19 tend to experience more severe symptoms (21) and higher risks of complications and death (22), and so focusing early vaccination efforts on groups with these vulnerabilities has the advantage of potentially preventing a larger number of serious cases and complications, and reducing the greatest source of demand for healthcare resources. In Qatar, 17 priority groups were established to determine vaccine administration. The highest priority was given to key workers including those involved in education, healthcare, and essential government services. Other priority groups included older adults.

## Methods

Data were obtained from three public sources. Daily vaccination numbers and information on the types of vaccine in use were obtained from data compiled by Our World in Data (23). Information regarding national policies on availability of the vaccine for people in specific high-risk groups was obtained from the Oxford COVID-19 Government Response Tracker (OxCGRT) (24). Additionally, aggregate records from a single mass vaccination site in Qatar were used to provide a detailed assessment of progress with respect to successful prioritization of high-risk patients. 21 June 2021 was selected as the end date for the study period, because that was the end of the mass vaccination campaign in Qatar. The study conformed to STROBE guidelines regarding conduct and reporting of a cross-sectional study (25). The study protocol was not pre-registered.

### Measures

#### Vaccination Start Date

The first date for which country-level mass vaccination data were reported, as indicated by the OWID data file, was defined as the vaccination start date. In some cases, this date may be later than the earliest date on which vaccinations were approved or administered, depending on individual governments’ reporting practices.

#### Cumulative Vaccination Totals

The OWID data tracks multiple measures of vaccination administration totals: doses administered, people vaccinated (i.e., the number of individuals receiving at least one vaccine dose), and people fully vaccinated (i.e., the number of individuals receiving a full course of vaccination, which may correspond to one or two doses depending on the type of vaccine received). Because a substantial number of countries had reported only the number of doses administered, and not the number of individuals vaccinated, all figures reported in this study refer to the number of vaccine doses administered.

Furthermore, the OWID data included both daily and cumulative dose administration totals based on government reports in each country. Since some countries did not report cumulative totals for each date, OWID also computed an interpolated daily vaccination total, based on a 7-day rolling average and computed according to the assumption that the vaccination rate changed at an equal rate across all days on which data were not reported. For this study, the daily total *per capita* vaccine administration was additionally computed by dividing this daily figure by the country population estimates used by the OWID. The reported (not interpolated) total was used in this study to indicate the total and *per capita* total vaccine administration at the end of the study period (21 June 2021).

#### Vaccination Milestone Dates

Vaccination pace was also indexed in this study by the number of days elapsed between the beginning of the mass vaccination program and the date on which certain levels of *per capita* vaccine administration were met. These milestones included administration of 5 doses per 100 population, 10 doses per 100 population, 25 doses per 100 population, and 50 doses per 100 population. For example, the days elapsed to administration of 5 doses per 100 was determined by subtracting the mass vaccination start date from the first date on which the cumulative total number of doses administered (as computed from the interpolated daily vaccination figures described above) was greater than or equal to 5.

#### Vaccine Administration Policy

The OxCGRT database includes a country-level measure of vaccination policy indicating the availability of the vaccine to specific groups at risk and to the general public. This numeric coding system ranges from 0 to 5, with 0 indicating no vaccine availability, 1 indicating availability to one high-risk population (key workers, clinically vulnerable populations other than older adults, or older adults), 2 indicating availability to two of these groups, 3 indicating availability of all three of these groups, 4 indicating availability to all three of these groups plus additional broad groups or age categories, and 5 indicating availability without limitations. The starting phase was defined as the availability phase according to national policy on the starting date of mass vaccine administration. This study also examined the number of days elapsed between phases, which was determined in a similar fashion to the vaccination milestone dates described above; for each country, the mass vaccination start date was subtracted from the date on which each successive phase began. Since many countries started with a policy defined as phase 2 or greater, the starting date for earlier phases is generally defined as missing. In a small number of cases, countries that started in a higher phase of vaccine availability later moved into more restrictive distribution. Those cases are reflected by higher elapsed days for earlier phases. In any cases in which a country moved into the same phase more than once, only the first instance is reflected in these figures. Additionally, these figures do not reflect cases in which availability may have been less restricted in certain sub-national divisions than as a matter of national policy.

## Results

Descriptive statistics for vaccination dates are presented in Table 1. Among GCC countries, Qatar was the first to begin mass vaccination, on 23 December 2020, with the UAE being the last to do so, on 7 January 2021. However, the UAE had achieved the highest rate of vaccine administration among GCC countries by 21 June 2021, with 147.9 doses per 100 population. The lowest rate was in Oman, with 15.8 doses administered per 100 population. Bahrain was the quickest to achieve several vaccination milestones, administering 5 doses per 100 population after 16 days, and 10 doses per 100 population after 35 days. The UAE, however, was somewhat quicker to reach milestones of 25 doses per 100 population (60 days), and 50 doses to 100 population (80 days). By these metrics, Qatar performed near the middle of the GCC nations, achieving an administration rate of 66.4 doses per 100 population by 12 May 2021, and having administered 5 doses per 100 population within 60 days.

**TABLE 1:** COVID-19 vaccination statistics for Gulf Cooperation Council, G7, and other Organization for Economic Cooperation and Development nations, Our World in Data 2020–2021.

Country	Vaccination begin date	Days to 5 doses per 100	Days to 10 doses per 100	Days to 25 doses per 100	Days to 50 doses per 100	Days to 100 doses per 100	Doses per 100 by 5/12/2021	Total doses by 6/21/2021
	GCC Countries							
Bahrain	12/25/2020	16	35	68	102	156	114.9	1,955,753
Kuwait	12/26/2020	59	81	125	172	—	51.6	2,202,753
Oman	12/27/2020	122	163	—	—	—	15.8	806,917
Qatar	12/23/2020	60	74	94	124	179	100.6	2,898,814
Saudi Arabia	12/17/2020	84	96	132	—	—	48.1	16,735,649
United Arab Emirates	11/25/2020	41	45	60	80	147	147.9	14,631,482
GCC Average [95% CI]		63.7 [25.4, 101.9]	82.3 [34.5, 130.2]	95.8 [55.5, 136.1]	119.5 [56.9, 182.1]	160.7 [119.7, 201.7]	79.8 [28.0, 131.7]	
	G7 Countries							
Canada	12/15/2020	75	95	122	154	—	86.4	32,598,064
France	1/5/2021	43	65	101	140	—	72.7	49,097,359
Germany	12/27/2020	49	73	110	143	—	80.7	67,584,932
Italy	12/27/2020	48	73	111	145	—	77.5	46,826,058
Japan	2/13/2021	89	102	123	—	—	29.8	37,622,424
United Kingdom	12/8/2020	37	46	71	110	179	110.0	74,638,083
United States	12/14/2020	38	51	81	113	—	95.3	318,576,441
G7 Average		54.1 [35.7, 72.6]	72.1 [52.9, 91.4]	102.7 [84.3, 121.2]	134.2 [115.1, 153.3]	—	78.9 [55.8, 102.0]	
	Other OECD Countries							
Australia	2/21/2021	52	75	—	—	—	24.8	6,316,375
Austria	12/27/2020	47	72	107	145	—	77.4	6,969,946
Belgium	12/28/2020	45	73	108	143	—	84.9	9,844,082
Chile	12/24/2020	47	51	74	92	160	112.6	21,529,168
Colombia	1/1/2021	96	120	162	—	—	30.1	15,298,930
Czech Republic	12/27/2020	53	75	116	155	—	68.8	7,367,706
Denmark	12/27/2020	39	61	107	144	—	79.4	4,598,488
Spain	12/27/2020	47	70	109	143	—	78.9	36,880,086
Estonia	12/27/2020	47	67	103	150	—	68.5	908,471
Finland	12/27/2020	50	70	112	151	—	71.3	3,950,537
Greece	12/27/2020	49	68	115	151	—	72.1	7,510,968
Hungary	12/26/2020	52	65	90	116	174	101.2	9,779,446
Ireland	1/4/2021	36	59	106	150	—	71.8	3,543,523
Iceland	12/29/2020	40	65	103	132	173	101.0	344,809
Israel	12/19/2020	9	12	26	39	78	123.1	10,659,925
Lithuania	12/27/2020	45	67	106	142	—	77.5	2,109,492
Luxembourg	12/28/2020	56	78	111	148	—	78.6	491,716
Latvia	12/28/2020	71	100	134	163	—	57.7	1,088,852
Mexico	12/24/2020	92	111	161	—	—	31.6	13,376,862
Netherlands	1/6/2021	45	65	104	140	—	78.1	13,376,602
Norway	12/18/2020	54	75	118	165	—	67.8	3,676,602
New Zealand	3/16/2021	43	64	—	—	—	20.7	998,266
Poland	12/30/2020	43	65	112	148	—	70.5	26,665,528
Portugal	12/27/2020	48	68	112	147	—	75.1	7,654,120
Slovakia	12/26/2020	48	71	116	160	—	61.5	3,357,289
Slovenia	12/26/2020	46	69	112	153	—	67.5	1,403,812
South Korea	2/26/2021	60	83	104		—	35.4	18,130,141
Sweden	12/27/2020	49	71	112	153	—	71.2	7,188,415
Switzerland	12/23/2020	48	70	117	150	—	79.2	6,851,588
Turkey	1/4/2021	43	54	111	167	—	51.7	43,629,179
OECD Average		50.8 [45.4, 56.2]	70.8 [64.6, 77.0]	108.2 [100.4, 116.0]	140.4 [131.2, 149.5]	152.8 [100.2, 205.4]	71.4 [63.4, 79.4]	

NOTES: GCC, Gulf Cooperation Council; OECD, Organization for Economic Cooperation and Development. Based on data obtained from Our World in Data (23).

Even greater heterogeneity in mass vaccination programs was observed among G7 nations, with start dates ranging from 14 December 2020 in the UK to 13 February 2021 in Japan. By 21 June 2021, the UK had achieved the highest rate of administration, with 110.0 doses given per 100 population, compared on the lower end with 29.8 doses per 100 population in Japan. The UK also set the fastest pace for vaccination, achieving 5 doses per 100 population in 37 days, and 50 doses per 100 population in 110 days.

Within the OECD as a whole (including the G7 countries described above), the best mass vaccination results were achieved in Israel, which reported 123.1 doses administered per 100 population by 21 June 2021, and which reached milestones of 5 doses per 100 population in 9 days, and 50 doses per 100 population in 39 days. Because of the high degree of variance within all of these groups of countries, no significant differences were detected in the means of any of these vaccination administration variables between the GCC, G7, and OECD.

**FIGURE 1:**
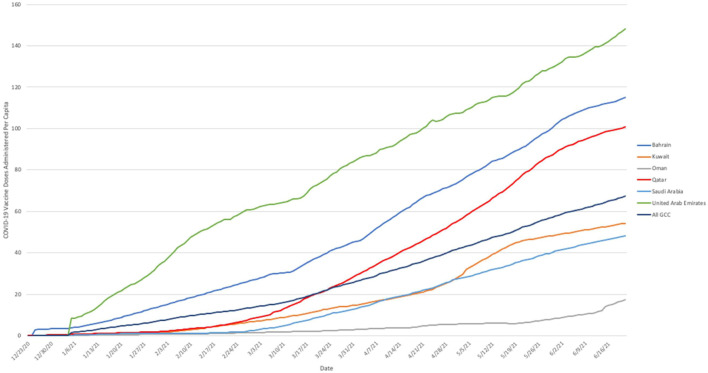
Cumulative number of COVID-19 vaccine doses administered *per capita* in Gulf Cooperation Council nations, 23 December 2020 to 21 June 2021. Note: Based on cumulative daily vaccination totals (where reported daily) and smoothed estimates of daily vaccine administration (where reported less often than daily). Source: Our World In Data (23) 2020–2021.

Progress in cumulative daily vaccination rates within the GCC is illustrated in Figure 1. Again, considerable heterogeneity among countries was evident. In Oman the rate of vaccination was relatively slow and remained fairly steady throughout the study period. In Qatar, Bahrain, and Saudi Arabia, the rate of progress increased notably during February and March 2021. In the UAE, the pattern of administration was more variable, with multiple apparent changes in rate, slowing at the beginning of February 2021 before increasing again in the middle of March 2021. Notably, the rate of vaccine administration in Qatar changed rapidly; after closely following a pattern similar to that seen in Kuwait until mid-February, considerably below the overall rate for the GCC as a whole, the vaccination rate increased rapidly over the following month so that it exceeded the GCC average by mid-March.

**FIGURE 2:**
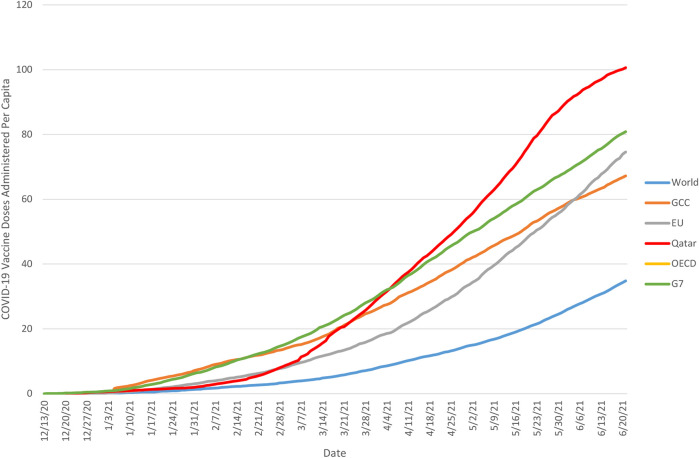
Cumulative number of COVID-19 vaccine doses administered *per capita* in Qatar and global benchmarks, 13 December 2020 to 21 June 2021. Note: Based on cumulative daily vaccination totals (where reported daily) and smoothed estimates of daily vaccine administration (where reported less often than daily). Source: Our World In Data (23) 2020–2021.

Figure 2 illustrates similarly illustrates the trajectory of cumulative daily vaccine administration in Qatar and the GCC as a whole in comparison to other global benchmarks, including the G7, OECD, EU, and the world as a whole. Once more, these trajectories demonstrated a great deal of variance, with the most rapid early progress demonstrated among the G7 nations, and global progress lagging considerably behind all of these groups. In the global context, the vaccination trajectory in Qatar is notable in that until late February 2021 its trajectory closely resembled that of the EU, trailing the GCC as a whole as well as the OECD and G7 nations, but changed rapidly after that point, exceeding progress in the GCC and OECD in mid-March, and achieving parity with the G7 by early April, and surpassing it later that month.

Table 2 describes the vaccination strategy of countries in the GCC, G7, and OECD in terms of availability to at-risk groups. In the GCC, most countries (Kuwait, Oman, Qatar, and Saudi Arabia) began their vaccination programs in “phase 3,” with availability to individuals in all three high risk categories based on occupation, age, and clinical risk. Availability was further expanded in Qatar and Saudi Arabia to include other broad risk categories. By contrast, the UAE began with availability to a single risk group, later expanding to additional groups, and Bahrain began with availability to the general public, only later scaling availability back to only members of at-risk groups. Vaccine access was generally much more restricted in the G7 and other OECD countries, with most limiting initial availability to one or two risk groups and opening up to others relatively slowly. Only the UK and Israel began their programs with availability to all three risk categories, and by 12 May 2021, only seven of the 37 OECD countries (18.9%) had extended vaccination eligibility to the general population.

**TABLE 2:** Mass vaccination availability phase progression by country for Gulf Cooperation Council, G7, and other Organization for Economic Cooperation and Development nations, Our World in Data 2020–2021.

Country	Start phase	Days to phase 2	Days to phase 3	Days to phase 4	Days to phase 5
GCC Countries					
Bahrain	5	—	80	144	—
Kuwait	3	—	—	141	—
Oman	3	—	—	149	—
Qatar	3	—	—	97	—
Saudi Arabia	3	—	—	31	84
United Arab Emirates	1	—	74	103	166
G7 Countries					
Canada	2	—	36	153	172
France	2	—	34	97	127
Germany	2	—	59	—	162
Italy	2	—	110	141	174
Japan	1	58	—	128	—
United Kingdom	3	—	—	99	—
United States	1	75	106	126	142
Other OECD Countries					
Australia	2	—	—	29	—
Austria	2	—	47	155	—
Belgium	1	22	102	147	172
Chile	1	47	130	152	—
Colombia	2	—	122	—	—
Czech Republic	2	—	79	147	—
Denmark	2	—	5	155	—
Spain	2	—	142	—	—
Estonia	1	14	71	127	141
Finland	1	9	64	156	—
Greece	1	80	149	163	—
Hungary	1	12	46	106	114
Ireland	1	7	68	167	—
Iceland	2	—	111	—	132
Israel	3	—	—	30	64
Lithuania	1	2	141	147	—
Luxembourg	2	64	65	99	128
Latvia	1	56	118	123	126
Mexico	1	53	128	—	—
Netherlands	1	24	44	150	—
Norway	1	20	85	161	—
New Zealand	1	59	—	42	—
Poland	2	—	92	120	125
Portugal	2	—	39	117	—
Slovakia	1	23	48	92	124
Slovenia	2	—	41	164	—
South Korea	2	—	25	—	—
Sweden	2	—	127	156	—
Switzerland	1	12	113	178	—
Turkey	1	24	77	—	—

Notes: Based on data obtained from the Oxford COVID-19 Government Response Tracker (OxCGRT) (24). Phase 1, vaccine availability limited to one high-risk population (key workers, clinically vulnerable populations other than older adults, or older adults). Phase 2, vaccine availability extended to two of the three high-risk groups listed above. Phase 3, vaccine availability in all three high-risk groups. Phase 4, vaccine availability in high-risk groups plus additional broad groups or age categories. Phase 5, universal vaccine availability. GCC, Gulf Cooperation Council; OECD, organization for economic cooperation and development.

In Qatar, the highest vaccination rates by 21 June 2021 had been achieved among older adults (97% partially or fully vaccinated, based on figures from a single representative mass vaccination site) and primary school teachers (94% by the same measures). By contrast, the priority group with the lowest vaccination level was Non-Qataris aged 35–39, of whom only 25% were vaccinated by the same date.

## Discussion

The clearest result of this study is the extreme degree of variance between countries in COVID-19 vaccine administration, in terms of the pace of mass vaccination, the policies used to determine vaccine availability, and the types of vaccine used. Even among the largely high income and developed economies that make up the GCC and OECD, vaccination rates by the end of the period covered by this study ranged from as low as 24.8 doses per 100 population to as high as 123.1 per 100 population. The strategy for making the vaccine available to members of various risk categories was also highly variable, with some countries moving rapidly to give the vaccine to multiple groups, and others focusing initially on a single key group (such as healthcare workers) and gradually expanding the scope of vaccination. While the first of these approaches appeared to be more popular among GCC countries than among OECD countries, there was again considerable variance within both groups.

A key point of focus of this study was to compare the mass vaccination program in Qatar with other regional and global benchmarks. In comparison to other GCC countries, Qatar was distinct in the degree to which its vaccination rate accelerated over the course of the study period. Until early February 2021, Qatar followed a trajectory that lagged behind the region as a whole, closely mirroring the trajectories of Kuwait, Saudi Arabia, and Oman. During early February, both Qatar and Kuwait began to accelerate their vaccine administration in comparison to Saudi Arabia and Oman. By late February, administration in Qatar increased at a markedly faster pace than Kuwait, so that by the end of the study period *per capita* dose administration in Qatar exceeded that of the GCC region as a whole. The Qatari approach contrasted with that taken in Bahrain and the UAE, which had more rapid rates of vaccination early in the mass vaccination process, in that Qatar used the Pfizer/BioNTech vaccine exclusively throughout the period covered by this study, whereas both Bahrain and the UAE had a broader range of vaccines at their disposal. It is plausible that this choice impacted the initial vaccination rate. However, given the lack of peer-reviewed efficacy data for the Sinopharm vaccines and persistent controversy regarding safety and data transparency for the Sputnik V vaccine (26), it remains unknown whether those possible gains in early vaccination rates will prove to be offset by increased risk and lower efficacy. The use of priority grouping in Qatar appears to have been at least broadly effective in delivering vaccinations to the highest risk groups, with very high proportions of older adults and key workers (particularly teachers).

In one sense, the observations made in this study underscore the importance of economic factors in supporting COVID-19 mass vaccination, since the relatively wealthy economies represented by the GCC and OECD have clearly performed better in this regard than the world average, with the wealthiest G7 economies performing the best as a group. Conversely, there is a great deal of heterogeneity among these wealthier countries as well, with some making relatively limited inroads in terms of vaccination in spite of their high GDPs. Factors including administrative decision-making processes, production and supply chain problems, and existing health infrastructure are likely to account for many of these points of variance.

Limitations of the present study include a lack of cross-national data regarding full and partial vaccination, and the necessarily interim nature of the analysis. Since all but one of the COVID-19 vaccines currently in use require administration of two doses, it is not possible to infer the complete vaccination rate from the number of doses administered. Although the OxCGRT database reports full and partial vaccination numbers where they are available, many national health authorities do not make this information public. Similarly, the data do not allow for conclusions to be drawn about the total number of members of vulnerable populations and the general population vaccinated, nor the number of doses of each type of vaccine administered. For example, it would be valuable to compare vaccination rates in the population of adults over age 65, to assess success in serving high-risk populations, but these data are not uniformly available across countries. Moreover, because of the dynamic nature of the global pandemic, the current study can provide only a snapshot of the development of mass vaccination in the relatively early phases. As countries begin to approach the level of vaccination required for herd immunity, patterns of mass vaccination will undoubtedly change. Additionally, it is critical to acknowledge that the interpretation of these data are inherently limited by the validity of the procedures used by the national agencies in reporting the original data. There are unknown degrees of inaccuracies and reporting latencies which differ between countries and across time, as a condition of data collection change. Thus all results must be interpreted with appropriate caution.

Nevertheless, this study demonstrates the heterogeneity of performance of even relatively wealthy countries in the process of mass vaccination. Given the unparalleled role of vaccination in the effort to end the global COVID-19 pandemic, understanding these differences is a vital step towards saving lives through improving the administration of these vaccines. The case of Qatar is illustrative of the fact that process improvements can result in substantive positive change in the pace of vaccination over the course of such a program. It is hoped that the insights presented in this study will help to guide future research in this area and will help to suggest avenues for quality improvement in ongoing and future mass vaccination efforts.

## Abbreviations

EU, European Union; GCC, Gulf Cooperation Council; GDP, Gross Domestic Product; OECD, Organization for Economic Cooperation and Development; OWID, Our World in Data; OxCGRT, Oxford COVID-19 Government Response Tracker; UAE, United Arab Emirates; UK, United Kingdom; WHO, World Health Organization.
